# PW02-013 - The role of IL6 and LPS in pathogenesis of TRAPS

**DOI:** 10.1186/1546-0096-11-S1-A153

**Published:** 2013-11-08

**Authors:** S Savic, L Ouboussad, LJ Dickie, CH Wong, R Doble, W Miriam, MF McDermott

**Affiliations:** 1 Clinical Immunology and Allergy, St James's University Hospital, Leeds, UK; 2 Experimental rheumatology, NIHR-Leeds musculoskeletal biomedical research unit, Leeds, UK; 3 University of Leeds, Leeds, UK; 4 Dermato-rheumatology research group, NIHR-Leeds musculoskeletal biomedical research unit, Leeds, UK

## Introduction

Tumour necrosis factor (TNF) receptor– associated periodic syndrome (TRAPS) is a hereditary autoinflammatory condition resulting from a range of mutations in the TNFRSF1A gene. It is characterised in part by recurrent episodes of inflammation, affecting connective tissues, and manifesting as migratory erysipelas, myalgia and serositis. A number of aberrant inflammatory responses have been described in this condition, including hyperresponsiveness to lipopolysaccharide (LPS). The microRNA mir155 has been implicated in mediating downstream pro-inflammatory response to LPS, and it has has also been implicated in the pathogenesis of rheumatoid arthritis (RA). Tocilizumab (anti-IL6 receptor antibody) has been reported to be an effective treatment in at least one published case of TRAPS.

## Objectives

To investigate the effects of LPS challenge on TRAPS fibroblasts and the roles of IL-6 and mir155 in this process.

## Methods

A patient with a T50M mutation who had previously failed to respond to etanercept and anakinra was consented to treatment with tocilizumab. Skin fibroblasts were obtained by biopsy and were propagated ex-vivo. The effects of LPS, IL-1 and TNF on IL-6 production were compared between patient’s fibroblasts and fibroblasts obtained from a healthy control (HC). TRAPS and HC fibroblasts, as well as synovial fibroblasts (SFs) from RA and osteoarthritis (OA) patients were then subjected to a graded LPS challenge. IL-6 levels were quantified from media supernatants using ELISA kits (BD Biosciences). mir155 levels were determined using qPCR from total RNA.

## Results

The patient’s fibroblasts showed greater production of IL-6 in response to LPS and IL-1 but not TNF when compared to HC. In the case of RA and OA SFs there was a dose dependent increase in mir155 levels, whilst, in the case of TRAPS fibroblasts, the response was not dose dependent but maximum levels were achieved even at the minimal dose of LPS (dose range 0.1 ng/l-100ng/l). The patient had a good clinical response to tocilizumab with a reduction in the symptoms, decreased steroid use and resolution of the biochemical markers of inflammation.

## Conclusion

Skin fibroblasts are capable of responding to LPS with production of mir-155 and IL-6. This response appears to be particularly enhanced in TRAPS fibroblasts, which may be one of the reasons why a number of clinical manifestations localise to the connective tissue. The ex-vivo experiments, showing hyperresponsiveness to LPS and the effective use tocilizumab in this patient would support a role for IL-6 in the pathogenesis of TRAPS.

## Disclosure of interest

None declared

